# Validation of the comprehensive feeding practices questionnaire in parents of preschool children in Brazil

**DOI:** 10.1186/s12889-016-3282-8

**Published:** 2016-07-19

**Authors:** Sarah Warkentin, Laís Amaral Mais, Maria do Rosário Dias de Oliveira Latorre, Susan Carnell, José Augusto de Aguiar Carrazedo Taddei

**Affiliations:** Department of Pediatrics, Discipline of Nutrology, Federal University of São Paulo (UNIFESP), Rua Loefgreen, 1647, CEP: 04040-032 São Paulo, SP Brazil; Department of Epidemiology, University of São Paulo (USP), School of Public Health, São Paulo, SP Brazil; Department of Psychiatry and Behavioral Sciences, Division of Child & Adolescent Psychiatry, Johns Hopkins University School of Medicine, Baltimore, MD USA

**Keywords:** Child nutrition, Feeding behavior, Food consumption, Preschool child, Validation studies, Parent-child relations

## Abstract

**Background:**

Recent national surveys in Brazil have demonstrated a decrease in the consumption of traditional food and a parallel increase in the consumption of ultra-processed food, which has contributed to a rise in obesity prevalence in all age groups. Environmental factors, especially familial factors, have a strong influence on the food intake of preschool children, and this has led to the development of psychometric scales to measure parents’ feeding practices. The aim of this study was to test the validity of a translated and adapted Comprehensive Feeding Practices Questionnaire in a sample of Brazilian preschool-aged children enrolled in private schools.

**Methods:**

A transcultural adaptation process was performed in order to develop a modified questionnaire (43 items). After piloting, the questionnaire was sent to parents, along with additional questions about family characteristics. Test-retest reliability was assessed in one of the schools. Factor analysis with oblique rotation was performed. Internal reliability was tested using Cronbach’s alpha and correlations between factors, discriminant validity using marker variables of child’s food intake, and convergent validity via correlations with parental perceptions of perceived responsibility for feeding and concern about the child’s weight were also performed.

**Results:**

The final sample consisted of 402 preschool children. Factor analysis resulted in a final questionnaire of 43 items distributed over 6 factors. Cronbach alpha values were adequate (0.74 to 0.88), between-factor correlations were low, and discriminant validity and convergent validity were acceptable.

**Conclusions:**

The modified CFPQ demonstrated significant internal reliability in this urban Brazilian sample. Scale validation within different cultures is essential for a more comprehensive understanding of parental feeding practices for preschoolers.

## Background

Childhood overweight is increasing worldwide, with prevalence of overweight in preschool children in Brazil rising considerably in the past decades, reaching 6.6 % in children with less than 5 years of age [1]. This negative outcome may be attributable to increased purchasing power within the Brazilian population, and parents’ valorization of overweight in this age group, due to previous food deprivation [2]. The extensive growth of the food industry, entry of multinational food companies, and expansion of media advertising of non-healthy foods – largely a result of national economy growth – may also play a role [3]. Certainly, studies have demonstrated a significant increase in availability and variety of products in Brazil, especially between 2002-2003 and 2008-2009, which is largely driven by increases in ultra-processed food consumption [4, 5].

Many of children’s eating habits are shaped at home by the influence of parents, making parent feeding the focus of a growing amount of research [6, 7]. Parents are a child’s first nutritional educators, playing a unique role in the development of eating habits during formative preschool years [8, 9]. Parental feeding practices can directly influence child’s eating behaviors and nutritional status [10], by creating different physical and social food environments. Various strategies used by parents to promote consumption of healthier food, such as pressure to eat and restriction, are known to be associated with poorer intake regulation and greater child food intake [11, 12]. Additionally, overweight status tends to persist from childhood into adulthood and an earlier onset and longer duration of obesity is associated with greater cardiovascular risk [13, 14]. Research focused on early feeding experiences is therefore crucial for prevention.

Measuring parental attitudes and behaviors can be difficult, since the underlying constructs are abstract and complex [15, 16]. In order to identify these subjective constructs, a number of tools have been developed. The development of culturally appropriate tools to identify factors relating to overweight and unhealthy eating habits in children is essential to fully understand ethnic differences [17]. However, only a few instruments have been validated to measure parental feeding practices across cultures, for example, the Child Feeding Questionnaire (CFQ) [18], a 31-item self-report questionnaire which has been widely used in different ethnic and cultural groups [17, 19–22], and the Comprehensive Feeding Practices Questionnaire (CFPQ) [23] which has been cross-validated in various age groups and countries such as the United States, France, Iran, New Zealand, Norway and Malaysia [23–28]. Only one questionnaire measuring parent feeding practices has been validated in Brazil – the Parent Mealtime Action Scale [29], which measures the most frequent actions used by parents during mealtime.

The CFPQ [23], a parent-report instrument, was designed to measure feeding practices of parents of 2-to-8-year-old children. It contains 49 items comprising 12 factors: ‘Encourage Balance and Variety’, ‘Environment’, ‘Involvement’, ‘Modeling’, ‘Monitoring’, ‘Teaching about Nutrition’, ‘Emotion Regulation’, ‘Food as Reward’, ‘Pressure’, ‘Child Control’, ‘Restriction for Health’ and ‘Restriction for Weight Control’. It was validated using a confirmatory factor analysis (CFA) as well as correlations between factors and with parent’s perceived responsibility for feeding and concerns about the child’s weight status (over- and underweight). We chose the CFPQ because it is one of the most recent questionnaires developed for this age group, and is based on two widely used questionnaires [18, 30] with the added advantages of including positive parental feeding practices, such as ‘Teaching about Nutrition’, and distinguishing between two types of Restriction – Restriction for Health, and Restriction for Weight Control. As the original tool was validated in a small sample of families, further investigation of the CFPQ is merited. Larger samples in homogeneous age groups allow not only control over potential confounders, but a better understanding of age-specific associations between parental feeding practices and child eating behaviors [10].

The aim of the current study was to test the validity of the translated and adapted CFPQ within a large sample of Brazilian parents of 2-to-5-year-olds enrolled in private schools. The preschool age group we chose is of particular interest, because pre-schoolers are more dependent on their parents than older children, and therefore food intake is highly affected by parents’ choices. Further, food neophobia (rejection of foods that are novel or unknown to the child) and fussiness (rejection of many different types of food, often resulting in inadequate dietary variety) [31] in relation to food are very common at this age, making parents’ attitudes and behaviors in relation to such behaviors important to study, in order to avoid negative nutritional consequences [32–34].

## Methods

### Overview

This study of Brazilian parents of 2-to-5-year-olds was composed of two phases: (1) Transcultural adaptation of the CFPQ, (2) Psychometric analysis including factor analysis and tests for internal consistency, factor correlations, discriminant and convergent validity, and test-retest reliability. To estimate sample size, we used the Gorsuch [35] criteria which suggest inclusion of at least five participants per question, or a minimum of 200 respondents. Since the CFPQ is composed of 49 questions, this estimation resulted in 245 individuals. Accounting for 10 % dropout, we therefore aimed to recruit 270 participants, in total.

For practical reasons, private schools in the cities of Campinas and São Paulo were contacted, via email or telephone, followed by a meeting with the schools’ headmaster and/or coordinator. Sixteen of the 48 contacted schools accepted the invitation to participate in the study. Two of these schools participated in a pilot study, and the remaining 14 participated in the main study. One of these 14 remaining schools also participated in a test-retest reliability procedure.

This research received ethical approval from the Federal University of Sao Paulo (UNIFESP) ethics committee.

### Phase 1: transcultural adaptation of CFPQ

Study researchers made contact with the corresponding author of the original scale asking for permission to translate and validate it into Portuguese, and agreement was obtained. Transcultural adaptation was initialized with the translation of the CFPQ into Portuguese by three pediatric nutrition researchers fluent in English who worked together by consensus to produce one translated instrument. A back translation was then made by a translator blind to the original version of the CFPQ. The same three researchers then translated the questionnaire into Portuguese a second time, in order to improve understanding and to reduce confusion regarding terminology [36].

After this step, the Portuguese version of the CFPQ was emailed to 11 dietitians, to evaluate its content validity. All the comments/suggestions were compiled and discussed in a 2-h expert panel session, resulting in a slightly modified version of the questionnaire (e.g. change in sentences order and replacement of specific words, such as ‘to regulate ’for ‘to control’, and ‘to discuss’ for ‘to talk’). Semantic equivalence of the new version was then tested in eleven parents of index children drawn at random from two classrooms within one of the selected schools, and some items were modified based on parent’s answers/ understanding (e.g. replacement of specific words, such as ‘to ensure’ for ‘to confirm’).

### Phase 2: validation of CFPQ

First, in order to expose any difficulties with questionnaire completion and increase data accuracy, we conducted a pilot study in two of the participating schools. Comments provided by parents during this study identified several aspects that needed to be changed to increase comprehension and specificity. When a parent showed confusion about an item, the researchers evaluated its content and changed sentence order or replaced specific words, if considered necessary, e.g. ‘to encourage’ for ‘to promote’, and ‘the food tastes good’ for ‘the food is tasty’.

After piloting, we conducted the main study. Survey packets including information letters, consent forms and self-administered questionnaires were left in each classroom at each participating school to be distributed to eligible children, with instructions to bring them home to be completed by one of the parents within two weeks. In one of the schools, the survey packets were administered and completed by parents before and after a parents and teachers’ meeting. Parent-reported anthropometric information for each child was obtained within the survey packet and BMI z-scores were calculated based on WHO data from 2006/2007, with cut-off values of < -3 z-score for ‘Extremely underweight’, ≥-3 and < -2 z-score for ‘Underweight’, ≥-2 and < +1 z-score for ‘Normal weight’, ≥ + 1 and < +2 z-score for ‘Overweight’, ≥ + 2 and < +3 z-score for ‘Obese’ and ≥ +3 z-score for ‘Extremely obese’ [37, 38].

All returned questionnaires were examined for inconsistencies and missing answers using a consistent protocol performed by two trained researchers. Parents were telephoned up to three times to resolve ambiguous responses. In case of missing phone numbers or parents not answering, the data were entered as ‘missing’ in the database. Any missing data in the CFPQ led to child exclusion from the dataset.

Finally, one of the participant schools was selected to examine test-retest reliability. After two weeks, respondent parents received the CFPQ, via school, to be answered again. This interval was chosen to limit the likelihood that feeding practices could have changed with child age, and to reduce the chance of participants responding primarily based on recall of their first set of answers [39].

### Statistical analysis

CFA was conducted on the 12-factor original model [23], and then, since the original factor structure was not replicated, exploratory factor analysis (EFA) was conducted. Since factors were hypothesized to correlate, oblique rotation (Promax) was used. Items were treated as ordinal and, to avoid over- or under-extraction of factors, we used the Kaiser criteria (the eigenvalues-greater-than-one-rule) [40] and required coefficients greater than 0.3 in the correlation matrix to retain a factor [41]. Scree plots were additionally examined. The internal consistency of items within each identified factor was tested using Cronbach’s alpha, with values higher than 0.70 considered acceptable [42]. The normality of each factor variable was tested using the Kolmogorov-Smirnov test, and, since distributions were not normal, we ran Spearman’s correlations to check for overlap between factors, with values r ≥ 0.85 considered indicative of strong overlap [41].

Discriminant validity was assessed by running Mann-Whitney’s tests comparing scale means between two groups (i.e. low and high) based on indices of children’s food intake accessed by a Food Frequency Questionnaire (FFQ). Since there was no FFQ validated in Brazil that met our criteria of being both brief and appropriate for the pre-school age group, the FFQ was developed specifically for this study. We included in the FFQ 13 categories of ultra-processed food items known to be associated with obesity, and focused on the foods most frequently consumed in the Brazilian population [43]. These were: fast-food (Sandwich/French fries/Pizza), instant noodles (Ramen noodle), soft drink, artificial juice (Powder mix/ in box/ concentrated), chips, sugary snacks (candy/ bubble-gum/ lollipop/ chocolate), breakfast cereal, chocolate milk (powder/ready to drink), crackers/biscuits/cakes with and without stuffing, ice-cream/popsicles, dairy desserts (pudding/petit suisse), processed meat (sausage/ham/turkey breast). Parents answered about child food intake in the last 7 days prior the interview using a 5 point scale (1 = No intake in the last 7 days; 2 = Ate 1 to 2 times in the last 7 days; 3 = Ate 3 to 4 times in the last 7 days; 4 = Ate 5 to 6 times in the last 7 days; 5 = Ate every day in the last 7 days). Considering the 13 food groups listed in this ultra-processed food category, we created one variable representing high or low consumption. To obtain mean values for ultra-processed food intake, we summed the 13 items and divided by the number of items. We also created a high or low consumption variable by using the group median as a cut-off for dichotomization with individuals with scores above the median classified as ‘high intake’ (189 (47.01 %)) and those with scores below the median classified as ‘low intake’ (213 (52.99 %)). Regarding child food intake differences across groups, we hypothesized that children with high intake of ultra-processed food would have greater negative feeding practices (e.g. use of food as a reward or to regulate emotion, pressure to eat), while those with low ultra-processed food intake would have greater positive feeding practices (e.g. modeling, monitoring and teaching about nutrition).

Following Musher-Eizenman & Holub [23], the convergent validity was assessed by running Spearman’s correlations between the proposed scales and three related attitude scales derived from Birch *et al.* [18]. ‘Concern about child overweight’ (three items) and ‘Perceived responsibility’ (three items) scales were taken directly from the CFQ. ‘Concern about child’s underweight’ (three items) was adapted from the CFQ by changing the words ‘overweight’ to ‘underweight’ and ‘diet’ to ‘eat more’, as recommended by Musher-Eizenman & Holub [18, 23]. We hypothesized that higher scores on parental concern about child weight would be associated with higher scores on negative feeding practices, and higher scores on parent’s perceived responsibility would be associated with higher scores on positive feeding practices. Cronbach’s alpha values for these scales in the current sample were as follows: ‘Concern about child overweight’ alpha = 0.80, ‘Concern about underweight’ alpha =0.86, ‘Perceived responsibility’ alpha = 0.76.

Finally, test-retest reliability was assessed by calculating intraclass correlation coefficients (ICC), for each factor of the proposed factor solution, with scales considered reliable if ICC values were greater than 0.40 [44]. Additionally, Bland-Altman’s graphs were created using MedCalc for Windows version 15.2.2 [45].

Data was entered twice and analyzed using Stata version 12.0 with the help of two trained assistant researchers [46].

## Results

### Participants

**Fig. 1:**
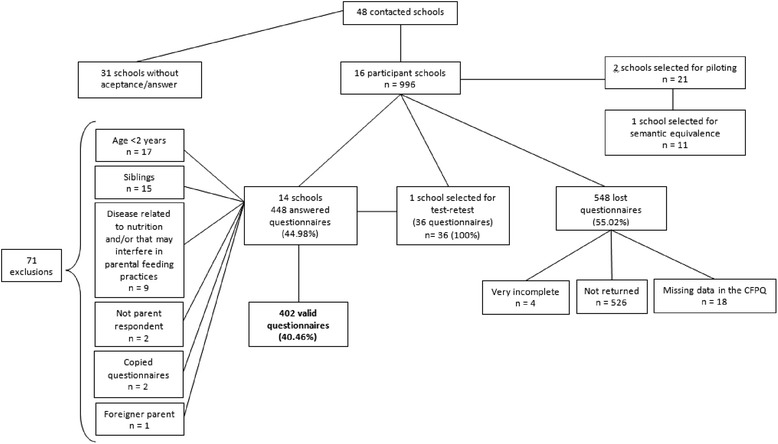
Participant flowchart of losses and exclusions

Of the total of 996 survey packets distributed, we received 448 questionnaires (45 %). Of the remaining 548, 526 were not returned, 18 had missing data on the CFPQ, and 4 had many incomplete answers to essential items concerning family characteristics. Of the completed 448 questionnaires, 15 were excluded due to participating children having siblings in the same age group (in case of siblings, the youngest child was included in the sample; in case of twins, the child whose name came first alphabetically was included), 17 for not being within the eligible age group, and nine for having diseases related to nutrition and/or other conditions that might interfere with parental feeding practices, such as lactose intolerance or cow’s milk protein allergy, cystic fibrosis, diabetes mellitus, down syndrome. We also excluded cases where the questionnaire was completed by individuals other than parents (*n* = 2), where parents had a mother-language other than Portuguese (*n* = 1) and one family where the parent had two children in the same age group and provided questionnaires with identical responses for each child, suggesting they may have assumed that the same answers applied for both children. Following exclusions, there were 402 valid questionnaires, producing an effective 40.4 % response rate (Fig. 1). For the test-retest reliability study we received 36 completed pairs from a total of 36 distributed questionnaires (100 % response rate).

Table 1 shows demographic and anthropometric characteristics of the final sample, which was 402 preschool children, 51.5 % male. Mean age was 3.1 years (+/- 0.78 SD). The majority of children were classified as normal weight (70.6 %). Most of the self-reported questionnaires were answered by mothers (93.5 %), whose mean age was 36.4 years. Almost all mothers completed college education (92.3 %) and their family’s income was considered high (60 % receiving more than 16 times the monthly minimum wage for Brazil, which is U$ 5148.32).

**Table 1:** Demographic and anthropometric characteristics of children (mean age 3.1 years (+/- 0.78, range 2-5y, *n* = 402)

Demographic and Anthropometric Characteristics	Category	n (%)
Child Sex	Male	207 (51.49)
	Female	195 (48.51)
Child BMI z-score	Extremely underweight (<-3)	7 (1.79)
	Underweight (≥-3 and < -2)	11 (2.81)
	Normal weight (≥-2 and < +1)	276 (70.59)
	Overweight (≥ + 1 and < +2)	63 (16.11)
	Obese (≥ + 2 and < +3)	18 (4.60)
	Extremely obese (≥ + 3)	16 (4.09)
Respondent	Mother	376 (93.53)
	Father	26 (6.47)
Maternal education	Middle school incomplete	1 (0.25)
	Middle school completed	0 (0.00)
	High school incomplete	1 (0.25)
	High school completed	6 (1.49)
	College incomplete	23 (5.72)
	College completed	371 (93.53)
Family’s income	Up to 5 times the minimum wage	24 (6.28)
	From 6 to 10 times the minimum wage	53 (13.87)
	From 11 to 15 times the minimum wage	69 (18.06)
	From 16 to 20 times the minimum wage	70 (18.32)
	More than 20 times the minimum wage	166 (43.42)
Maternal BMI	Underweight	15 (3.79)
	Normal weight	286 (72.22)
	Overweight	76 (19.19)
	Obese	19 (4.80)

*BMI* Body Mass Index. Brazilian Minimum wage: R$724.00 (US$321.77) in 2014 (Act n.8.166 from 23th Dec 2013)

### Factor analysis

After excluding negative correlations, EFA revealed a 6-factor structure. Five items with factor loadings lower than 0.3 were excluded. These items included the entire ‘Child Control’ factor. Two further items were also excluded, due to negative factor loadings (item 14 (‘*I keep a lot of snack food (potato chips, Doritos, cheese puffs) in my house’)* and item 16 (*‘I keep a lot of sweets (candy, ice cream, cake, pies, pastries) in my house’*). This resulted in a final questionnaire with 43 items contributing to six factors: ‘Healthy Eating Guidance’ (16 items), ‘Monitoring’ (5 items), ‘Restriction for Weight Control’ (7 items), ‘Restriction for Health’ (5 items), ‘Emotion Regulation/Food as Reward’ (6 items), and ‘Pressure’ (4 items) (see Appendix 1: Table 4).

Derived factors were as follows:

- *Healthy Eating Guidance:* This factor describes parents’ facilitation of a healthy eating environment, including teaching, modeling and child’s involvement in food intake. It is composed of the original ‘Encourage Balance and Variety’, ‘Environment’, ‘Involvement’, ‘Modeling’ and ‘Teaching about Nutrition’ factors.
- *Monitoring*: This factor captures the degree to which the parent keeps track of their child’s consumption of unhealthy foods, and replicates the entire original ‘Monitoring’ factor.
- *Restriction for Weight Control*: This factor assesses how much a parent restricts her/his child’s food intake in order to limit weight gain. This factor almost replicates the entire original ‘Restriction for Weight Control’ factor. However, one item (*‘I have to be sure that my child does not eat too many high-fat foods’*) loaded on the ‘Restriction for Health’ factor.
- *Restriction for Health*: This factor also measures how much a parent restricts child’s food intake, but with the focus on healthy eating rather than body weight. This factor replicates the entire original ‘Restriction for Health’ factor, with the addition of one question of the original ‘Restriction for Weight Control’ factor, as mentioned above.
- *Emotion Regulation/Food as Reward*: This factor determines how much a parent uses food as reward for desired behavior in their child, or to regulate emotion. This factor is composed of the original ‘Emotion Regulation’ and ‘Food and Reward’ factors.
- *Pressure*: This factor investigates how much a parent pressures the child to eat, and replicates the entire original ‘Pressure’ factor.

**Fig. 2:**
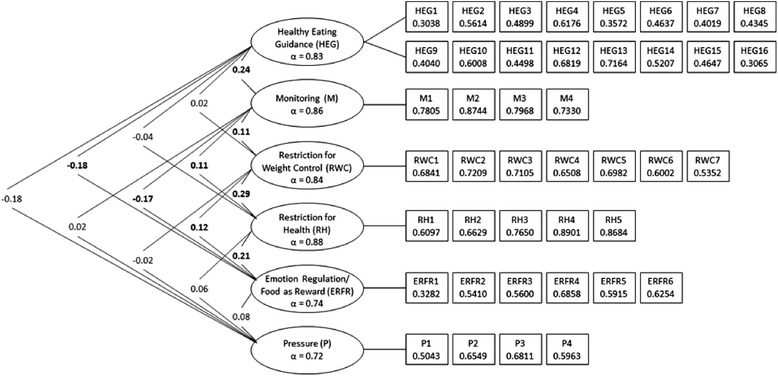
Spearman’s correlations between factors, Cronbach’s alpha for each sub-scale, and factor loadings for each item. *Note.* Values on the left side of the figure are correlations (Spearman’s rho), with significant correlations (*p* < 0.05) in bold. Values in ovals are Cronbach’s alpha (α) for each derived sub-scale (all over 0.70). Values in boxes give factor loadings from EFA (all over 0.30)

Figure 2 indicates Spearman’s correlations (rho) between newly emerging factors, Cronbach’s alphas (α) for each factor, and factor loadings for each item. The highest correlations were observed between the ‘Restriction for Health’ and ‘Restriction for Weight Control’ factors (*rho* = 0.29, *p* < 0.05). ‘Emotion Regulation/Food as Reward’ and ‘Healthy Eating Guidance’ were negatively correlated. However, all correlations were lower than 0.85, indicating the absence of significant factor overlap.

Assessment of test-retest reliability demonstrated ICC values for the proposed scale ranging from 0.42 to 0.81. Bland-Altman’s graphs (not presented) showed a random distribution of observations.

### Discriminant and convergent validity

Discriminant validity between scales and child’s ultra-processed food intake is described in Table 2. ‘Healthy Eating Guidance’ (*p* = 0.045), ‘Monitoring’ (*p* < 0.001), ‘Restriction for Health’ (*p* = 0.008) and ‘Emotion Regulation/Food as Reward’ (*p* = 0.003) factors were able to differentiate children with low and high ultra-processed food intake. 'Restriction for Weight Control’ and ‘Pressure’ were not able to identify differences between child’s intake.

**Table 2:** Discriminant validity for the modified CFPQ using ultra-processed food intake

Factors	Ultra-processed Food		p*
	Low Intake	High Intake	
	M (SD)	M (SD)	
Proposed Scale			
Healthy Eating Guidance	4.43 (0.41)	4.33 (0.46)	0.045
Monitoring	4.63 (0.62)	4.37 (0.76)	<0.001
Restriction for Weight Control	2.10 (0.86)	2.14 (0.94)	0.828
Restriction for Health	3.48 (1.20)	3.80 (1.06)	0.008
Emotion Regulation/Food as Reward	1.56 (0.59)	1.77 (0.69)	0.003
Pressure	3.26 (0.97)	3.39 (0.90)	0.349

*M* means, *SD* standard deviation, *p p* value.*Mann-Whitney’s test. Low ultra-processed food intake < =1.69 (Median of the 13 food groups) and High ultra-processed food intake >1.69 (Median of the 13 food groups). Response options: 1 = No intake in the last 7 days; 2 = Ate 1 to 2 times in the last 7 days; 3 = Ate 3 to 4 times in the last 7 days; 4 = Ate 5 to 6 times in the last 7 days; 5 = Ate every day in the last 7 days

Table 3 shows the correlation coefficients between each factor and three parental attitudes (perceived responsibility for feeding, concern about child’s under- and overweight). Parental responsibility for feeding was positively associated with ‘Healthy Eating Guidance’ and ‘Monitoring’ (*rho* = 0.20 and *rho* = 0.12, (*p* < 0.05), respectively). Greater use of ‘Pressure’ was associated with parent’s concern about child’s underweight (*rho* = 0.22, *p* < 0.001) and parents more concerned about child’s overweight tended to use more 'Restriction' (motivated for either weight control or health) (*rho* = 0.36 and *rho* = 0.20, (*p* < 0.001), respectively).

**Table 3:** Convergent validity between factors within the proposed scale, and parents’ perceived responsibility for feeding and concern about over- and underweight

Factors	Perceived Responsibility for Feeding	Concern about Overweight	Concern about Underweight
	*rho* (*p*)	*rho* (*p*)	*rho* (*p*)
Proposed Scale			
Healthy Eating Guidance	0.20 (<0.001)	0.00 (0.953)	0.01 (0.833)
Monitoring	0.12 (0.014)	0.05 (0.342)	0.03 (0.602)
Restriction for Weight Control	-0.06 (0.272)	0.36 (<0.001)	0.02 (0.715)
Restriction for Health	0.08 (0.101)	0.20 (<0.001)	0.06 (0.214)
Emotion Regulation/Food as Reward	-0.07 (0.137)	0.04 (0.460)	0.05 (0.342)
Pressure	0.01 (0.871)	-0.02 (0.629)	0.22 (<0.001)

*rho* correlation coefficient, *p p* value, Spearman’s test

## Discussion

The aim of this study was to test the validity and reliability of a Portuguese version of the CFPQ within a large sample of Brazilian parents of 2-to-5-year-olds. The translation, adaptation and factor analysis produced a final questionnaire of 43 items distributed over six factors, with good Cronbach’s alpha values, low between-factor correlations, and acceptable discriminant validity and convergent validity. Consistent with various attempts to validate the CFPQ in other languages and countries, such as France, Norway, Iran, New Zealand and Malaysia [24–28] we were unable to confirm the original CFPQ structure. Notably, the New Zealand study, which used 1013 parents of 4-to-8-year-olds, obtained a version of the instrument which was comparable to ours, composed of five factors and 32 items. The other validation studies, though, derived almost or the same number of factors as the original scale (Norway: 10 factors, Iran: 12 factors, Malaysia: 12 factors). However, it is important to note that there were significant methodological differences between ours and the other studies. For example, most did not perform full transcultural adaptation, which involves translation and back-translation of the instrument into target language, test of internal consistency, test-retest reliability, convergent and discriminant validity, and only the Iranian version of the instrument was validated in preschool aged children (3-to-5 years old) [25]. The present study is the first to assess the validity and reliability of the CFPQ for Brazilian families with preschool-aged children.

As expected due to cultural and social differences [47], we identified some important differences between our and the original scale structure [23]. In our model, four factors (‘Encourage Balance and Variety’, ‘Environment’, ‘Modeling’ and ‘Teaching about Nutrition’) combined together to form one single factor which we named ‘Healthy Eating Guidance’. A further combination obtained for ‘Emotion Regulation’ and ‘Food as Reward’ factors, resulted in one factor. Item 39 (“I have to be sure that my child does not eat too many high-fat foods”) from the original ‘Restriction for Weight Control’ factor loaded onto the new ’Restriction for Health’ factor. Factor combination results in a loss of the ability to detect more specific behaviors. However, we can conclude that these behaviors tend to occur together and therefore summing all the practices is likely to produce a more robust single factor [26]. In general, questionnaire structures composed of fewer factors provide a more parsimonious solution for statistical analysis and may result in more interpretable outcomes [41].

It was also notable that the entire ‘Child Control’ factor was excluded in our factor solution. The lack of coherence of the Child Control items could have been due to a number of reasons. For example, our age group (2-to-5 years old) was young compared to that used for the original Musher-Eizenman study (2-to-8 years old) [20], so items describing child control may have made less sense, leading to individual variability in how parents interpreted each question. In addition, ‘Child Control’ is a complex factor, including questions that could be interpreted as representing both negative and positive practices. In support of this explanation, in our sample, the Child Control items actually loaded on three different factors, although all factor loadings were <0.3, leading to exclusion. Cultural factors such as Brazilian parents conceiving the parent-child relationship differently than parents from other countries, may also have led to variability in interpretation of items on this particular scale; such variability would not necessarily be picked up in the pilot study, which simply checked that each parent felt they understood the meaning of each item. So, we suggest that more studies are necessary to clarify Brazilian parental feeding practices around child control [26]. Additionally, items 14 (“I keep a lot of snack food (potato chips, Doritos, Cheese puffs) in my house”) and 16 (“I keep a lot of sweets (candy, ice cream, cake, pies, pastries) in my house”) from the ‘Environment’ factor did not load onto any of the factors, likely due to variability in item comprehension, for example, the expression “a lot” of snack food/ sweets” may have been interpreted in different ways by different parents. Notably, only the CFPQ validation study in school-aged children from Malaysia [28] replicated the ‘Healthy Eating Guidance’ factor, suggesting more research into this construct is needed. Interestingly, the proposed scale demonstrated higher Cronbach’s alphas values (0.72 to 0.88) than the original scale (0.58 to 0.81) [23], suggesting improved internal consistency.

Results of the correlation analysis between the CFPQ factors revealed, overall, low correlations (Fig. 2), which were also found in the original scale [23]. The highest positive correlation was found between ‘Restriction for Weight Control’ and ‘Restriction for Health’ factors, which was expected due to each scale representing conceptually close constructs. Indeed, Musher-Eizenman & Holub suggest that parents may not spontaneously introspect a difference between restriction motivated by weight or by health reasons [23]. Notably, a positive correlation was also observed between ‘Healthy Eating Guidance’ and ‘Monitoring’ factors (*rho* = 0.24, *p* < 0.05), each of which measure healthier eating and may therefore be recommended in tandem to parents. A high *rho*-value between ‘Healthy Eating Guidance and ‘Monitoring’ was also observed in another CFPQ validation study [26].

‘Healthy Eating Guidance’ and ‘Emotion Regulation/Food as Reward’ were negatively correlated, which was also expected since these factors measure two potentially opposing practices: a positive, health related feeding practice, and a negative, coercive feeding practice, respectively. Using food as a tool to influence children’s emotions is associated with overconsumption following an emotion-induction procedure [48]. Practices such as using food (usually sweets) as a reward may make the ‘reward’ food more desirable and the ‘means’ food that the child is rewarded for eating (usually vegetables) less desirable [49–52].

Discriminant validity analyses confirmed the hypothesis that high intake of ultra-processed food would be related to negative feeding practices and low ultra-processed food intake to positive feeding practices. The new sub-scales ‘Healthy Eating Guidance’, ‘Monitoring’, ‘Restriction for Health’ and ‘Emotion Regulation/Food as Reward’ were able to significantly discriminate child’s ultra-processed food intake. Specifically, the frequency of ultra-processed food intake was higher in children whose parents used more restriction for health reasons, or when parents used food as a reward or to influence their child’s emotional state. Controlling feeding practices such as restriction and using food as reward have been linked to negative child outcomes such as overeating, which can result in excessive weight gain [47, 48, 52, 53]. Causal relationships are difficult to prove, but these associations might exist because coercive feeding practices undermine child’s ability to respond to their own internal cues of hunger and satiety [49, 53] suggesting that they should be discouraged in parents [51]. Alternatively, children’s unhealthy eating habits could lead parents to respond by engaging in restrictive and controlling practices [8]. ‘Healthy Eating Guidance’ and ‘Monitoring’, on the other hand, were associated with lower ultra-processed food intake. These positive practices are the most commonly reported goals of parents in previous studies [52], and are essential for young children, who are unable to choose a balanced and healthy meal without parental guidance and monitoring [54].

Analyses of convergent validity between scales and parents’ attitudes revealed that the hypotheses that higher scores on parental concern about child weight would be associated with higher scores on negative feeding practices, and higher scores on parent’s perceived responsibility would be associated with higher scores on positive feeding practices, were both true. The six factors from the proposed scale correlated with attitudes as expected based on previous studies [23, 47]. Greater parental responsibility for feeding scores were associated with higher ‘Healthy Eating Guidance’ and ‘Monitoring’ scores, greater concern about child overweight was associated with greater restriction, for either health or weight control, and greater concern about child underweight was associated with greater pressure to eat. One possible explanation is that parents consciously restrict child’s food intake when they perceive him/her to be overweight [18, 47, 55], while parents who are worried about their child being underweight pressure them to eat more at mealtimes [9, 55, 56].

Strengths of this study include the large number of parent-child dyads and the demonstration of test-retest reliability, which was not reported for the original validation study. In addition to being the first validation of CFPQ in Brazilian preschoolers, it is, to our knowledge, the first validation study of the CFPQ in any large preschool sample. The emergence of a scale with fewer items is a slight advantage, and the reduced factor number (from 12 to 6) is a significant advantage as it leads to more parsimonious modeling and potentially requires fewer respondents.

A limitation is that the generalizability of these results may be limited to Brazilian families with relatively high education and income. However this feature of the sample may have increased the ability of parents to understand the self-report questionnaire. Although the majority of the population in Brazil is low in income, high income individuals make up a significant minority. Compared to low income parents, whose purchasing power is relatively constrained, high income parents are more able to buy and offer whatever they wish to their children, potentially increasing the likelihood of purchasing less healthy foods. In fact, in contrast to the negative socioeconomic gradient that is usually observed in US populations, evidence suggests that the prevalence of obesity among preschool children in Brazil is higher in those with higher socioeconomic status [2]. Nevertheless, social desirability bias may have been present, with parents potentially feeling pressure to report a higher rate of healthy feeding practices [26, 47]; this is a challenge for most parent feeding studies [49].

## Conclusions

Psychometric properties of an adapted Portuguese version of the CFPQ were found to be equivalent or improved compared to the original questionnaire in this large sample of parents of 2-5 year olds in Brazil. Distinctions were expected, and found, since the original validation was conducted with a different age group (2-to-8-year-olds) and within an American population. In addition to understanding the influence of feeding practices *within* countries, such as Brazil, it is important to compare parental feeding practices *across* countries. Since some of our factors are identical or very similar to the original CFPQ factor solution, comparisons of scores on these derived sub-scales might be validly compared across populations. When factors appear to differ across countries, these different factor structures should be considered when conducting any analysis of between-country differences. Within-country effects of ethnicity, culture and environment might also be examined using an instrument specifically validated for the country in question, e.g. the Brazilian CFPQ. The fact that the factor structure of the CFPQ has been quite robust across several different validation populations, including our own, suggests that there are indeed many commonalities in the structure of feeding practices, which would facilitate cross-country comparisons.

The slightly modified scale reported here is valid and reliable for specifically assessing parental feeding practices in families with 2-to-5-year-olds in a Brazilian setting. Research on parent feeding practices in this age group is essential, because children are transitioning to the family diet, are learning much about food, and eating habits occur in the first years of life. Parental and home interventions at this age therefore hold great potential to limit long-term risks of conditions such as obesity and eating disorders.

## Abbreviations

BMI, body mass index; CFPQ, comprehensive feeding practices questionnaire; CFQ, child feeding questionnaire; CFQ, confirmatory factor analysis; EFA, exploratory factor analysis; FFQ, food frequency questionnaire; ICC. intraclass correlation coeficients; WHO, World Health Organization

## Appendix 1

**Table 4:** Factors and items from the proposed scale and original factors

Factors and Items from the proposed scale	Original Factors [19]
Healthy Eating Guidance (HEG)	
HEG1. Do you encourage this child to eat healthy foods before unhealthy ones? ^a^	Encourage Balance and Variety
HEG2. I encourage my child to try new foods.^b^	Encourage Balance and Variety
HEG3. I tell my child that healthy food tastes good.^b^	Encourage Balance and Variety
HEG4. I encourage my child to eat a variety of foods.^b^	Encourage Balance and Variety
HEG5. Most of the food I keep in the house is healthy.^b^	Environment
HEG6. A variety of healthy foods are available to my child at each meal served at home.^b^	Environment
HEG7. I involve my child in planning family meals.^b^	Involvement
HEG8. I allow my child to help prepare family meals.^b^	Involvement
HEG9. I encourage my child to participate in grocery shopping.^b^	Involvement
HEG10. I model healthy eating for my child by eating healthy foods myself.^b^	Modeling
HEG11. I try to eat healthy foods in front of my child, even if they are not my favorite.^b^	Modeling
HEG12. I try to show enthusiasm about eating healthy foods.^b^	Modeling
HEG13. I show my child how much I enjoy eating healthy foods.^b^	Modeling
HEG14. I discuss with my child why it’s important to eat healthy foods.^b^	Teaching about Nutrition
HEG15. I discuss with my child the nutritional value of foods.^b^	Teaching about Nutrition
HEG16. I tell my child what to eat and what not to eat without explanation.**R** ^b^	Teaching about Nutrition
Monitoring (M)	
M1. How much do you keep track of the sweets (candy, ice cream, cake, pies, pastries) that your child eats?^a^	Monitoring
M2. How much do you keep track of the snack food (potato chips, Doritos, cheese puffs) that your child eats?^a^	Monitoring
M3. How much do you keep track of the high-fat foods that your child eats?^a^	Monitoring
M4. How much do you keep track of the sugary drinks (soda/pop, kool-aid) this child drinks?^a^	Monitoring
Restriction for Weight Control (RWC)	
RWC1. I encourage my child to eat less so he/she won’t get fat.^b^	Restriction for Weight Control
RWC2. I give my child small helpings at meals to control his/her weight.^b^	Restriction for Weight Control
RWC3. If my child eats more than usual at one meal, I try to restrict his/her eating at the next meal.^b^	Restriction for Weight Control
RWC4. I restrict the food my child eats that might make him/her fat.^b^	Restriction for Weight Control
RWC5. There are certain foods my child shouldn’t eat because they will make him/her fat.^b^	Restriction for Weight Control
RWC6. I don’t allow my child to eat between meals because I don’t want him/her to get fat.^b^	Restriction for Weight Control
RWC7. I often put my child on a diet to control his/her weight.^b^	Restriction for Weight Control
Restriction for Health (RH)	
RH1. If I did not guide or regulate my child’s eating, s/he would eat too much of his/her favorite foods.^b^	Restriction for Health
RH2. If I did not guide or regulate my child’s eating, he/she would eat too many junk foods.^b^	Restriction for Health
RH3. I have to be sure that my child does not eat too much of his/her favorite foods.^b^	Restriction for Health
RH4. I have to be sure that my child does not eat too many sweets (candy, ice cream, cake, or pastries).^b^	Restriction for Health
RH5. I have to be sure that my child does not eat too many high-fat foods.^b^	Restriction for Weight Control
Emotion Regulation/ Food as Reward (ERFR)	
ERFR1. When this child gets fussy, is giving him/her something to eat or drink the first thing you do?^a^	Emotion Regulation
ERFR2. Do you give this child something to eat or drink if s/he is bored even if you think s/he is not hungry?^a^	Emotion Regulation
ERFR3. Do you give this child something to eat or drink if s/he is upset even if you think s/he is not hungry?^a^	Emotion Regulation
ERFR4. I offer sweets (candy, ice cream, cake, pastries) to my child as a reward for good behavior.^b^	Food as Reward
ERFR5. I withhold sweets/dessert from my child in response to bad behavior.^b^	Food as Reward
ERFR6. I offer my child his/her favorite foods in exchange for good behavior.^b^	Food as Reward
Pressure (P)	
P1. My child should always eat all of the food on his/her plate.^b^	Pressure
P2. If my child says, “I’m not hungry,” I try to get him/her to eat anyway.^b^	Pressure
P3. If my child eats only a small helping, I try to get him/her to eat more.^b^	Pressure
P4. When he/she says he/she is finished eating, I try to get my child to eat one more (two more, etc.) bites of food.^b^	Pressure
Excluded Items	
1. Do you let your child eat whatever s/he wants?^a^	Child Control
2. At dinner, do you let this child choose the foods s/he wants from what is served?^a^	Child Control
3. If this child does not like what is being served, do you make something else?^a^	Child Control
4. Do you allow this child to eat snacks whenever s/he wants?^a^	Child Control
5. Do you allow this child to leave the table when s/he is full, even if your family is not done eating?^a^	Child Control
14. I keep a lot of snack food (potato chips, Doritos, cheese puffs) in my house.^b^	Environment
16. I keep a lot of sweets (candy, ice cream, cake, pies, pastries) in my house.^b^	Environment

Note. Numbers correspond to the original items’ order in: Musher-Eizenman D, Holub S. Comprehensive Feeding Practices Questionnaire: validation of a new measure of parental feeding practices. J Pediatr Psychol. 2007;32(8):960-72 [23]. Items marked with a ^a^ utilize a 5-point-likert scale “never, rarely, sometimes, mostly, always”. Items marked with ^b^ utilize a 5-point-likert scale “disagree, slightly disagree, neutral, slightly agree, agree”. Items marked with an **R** were reverse coded
